# Nontraditional systems in aging research: an update

**DOI:** 10.1007/s00018-020-03658-w

**Published:** 2020-10-09

**Authors:** Justyna Mikuła-Pietrasik, Martyna Pakuła, Małgorzata Markowska, Paweł Uruski, Ludwina Szczepaniak-Chicheł, Andrzej Tykarski, Krzysztof Książek

**Affiliations:** 1 Department of Pathophysiology of Ageing and Civilization Diseases, Poznań University of Medical Sciences, Długa 1/2 Str., 61-848 Poznań, Poland; 2 Department of Hypertensiology, Poznań University of Medical Sciences, Długa 1/2 Str., 61-848 Poznań, Poland

**Keywords:** Aging, Immortal animals, Longevity, Long-lived species, Systems of aging

## Abstract

Research on the evolutionary and mechanistic aspects of aging and longevity has a reductionist nature, as the majority of knowledge originates from experiments on a relatively small number of systems and species. Good examples are the studies on the cellular, molecular, and genetic attributes of aging (senescence) that are primarily based on a narrow group of somatic cells, especially fibroblasts. Research on aging and/or longevity at the organismal level is dominated, in turn, by experiments on *Drosophila melanogaster*, worms (*Caenorhabditis elegans*), yeast (*Saccharomyces cerevisiae*), and higher organisms such as mice and humans. Other systems of aging, though numerous, constitute the minority. In this review, we collected and discussed a plethora of up-to-date findings about studies of aging, longevity, and sometimes even immortality in several valuable but less frequently used systems, including bacteria (*Caulobacter crescentus*, *Escherichia coli*), invertebrates (*Turritopsis dohrnii, Hydra sp., Arctica islandica*), fishes (*Nothobranchius sp.,* Greenland shark), reptiles (giant tortoise), mammals (blind mole rats, naked mole rats, bats, elephants, killer whale), and even 3D organoids, to prove that they offer biogerontologists as much as the more conventional tools. At the same time, the diversified knowledge gained owing to research on those species may help to reconsider aging from a broader perspective, which should translate into a better understanding of this tremendously complex and clearly system-specific phenomenon.

## Introduction

Aging is a phenomenon that may be considered from different perspectives (evolution, mechanisms) and at different levels of organization (populations, individuals, tissues/organs, cells, macromolecules). According to the book *Evolutionary Biology of Aging*, this term refers to “a persistent decline in the age-specific fitness components of an organism due to internal physiological deterioration” [1]. Clearly, diversified nature of aging was the prime reason of the unpredictable trajectory of research on this process. The natural history of investigations in this area is full of ground-breaking discoveries, whose number probably equals the number of empty routs and shattered hopes. For example, when we talk about aging at a cellular level (called *senescence*), an original idea of Alexis Carrel that somatic cells, and plausibly human beings, are intrinsically immortal [2] has been eradicated by the well-grounded observation by Leonard Hayflick and Paul Moorhead that normal cells have a predetermined number of population doublings and eventually degenerate, senesce, and die [3]. A paradigm in which cancer cells avoid Hayflick’s limit (do not senesce) and proliferate indefinitely [4] was crushed when therapy-induced [5], and later, spontaneous senescence of these cells was documented [6]. One of the most acknowledged theories of aging, the free radical hypothesis, also has its own ups and downs [7]. For example, research on invertebrates (*Drosophila melanogaster*) showed that flies with a deficiency in the antioxidative enzyme superoxide dismutase (SOD) display increased oxidative stress and a shortened life span suggesting that the magnitude of oxidative stress is positively correlated with the rate of aging [8]. Similar reaction was observed in *Saccharomyces cerevisiae* in which deletion of the gene for SOD accelerated chronological aging and overexpression of the enzyme increases the life span [9, 10]. When similar approach was tested in case of mammals (*Mus musculus*), the animals with overexpressed SOD displayed decreased oxidative stress, but their life span remained unchanged, which partly challenged the role of this agent in their aging [11]. The results obtained in invertebrates that did not translate to higher organisms do not mean that such the classic systems did not provide valuable and more universal findings. For example, Rose and Charlesworth evidenced the correctness of evolutionary theory of antagonistic pleiotropy using the selection experiments on *D. melanogaster* [12].

All these examples are listed to argue that despite more than a century of fruitful history of experimental gerontology, we are still uncertain about why and how we age. Or, as George Sacher asked, why do we live as long as we do [13]? In our opinion, there is another critical question to ask: do we sufficiently and cleverly use all available systems of aging and longevity and garner from them everything worthy of being learned? When we use a term a system, we think about a biological system (from unicellular to multicellular) whose analysis (starting from observations ending on advanced molecular tests) may provide answers to fundamental questions about evolutionary and mechanistic reasons of age-related morphological and functional deterioration leading to increased probability of death.

The question asked above stems from the fact that the majority of the knowledge that we collected regarding mechanistic and evolutionary aspects of aging derives from a somewhat limited number of experimental systems. For example, current views on cellular senescence primarily stem from research on a few cell types, particularly fibroblasts. Knowledge about organismal aging is mainly based on investigations on invertebrates (*Saccharomyces cerevisiae*, *Caenorhabditis elegans*, *Drosophila melanogaster*) and mammals (*Mus musculus*, *Homo sapiens*). The spectrum of valuable systems for studying aging is, however, much more extensive, which allows us to look at this phenomenon using a holistic rather than a reductionist approach (Table 1). The same applies for potential benefits that could be extracted from studies on less popular systems. This review is aimed at providing up-to-date information regarding aging and its two permutations: longevity (an extraordinary lifespan) and immortality (unending existence) in numerous less conventional systems, which are complementary and sometimes even ahead of the advances reached using more traditional tools. We call longevity as a permutation of aging, because there are some conceptual challenges that jeopardize a simple classification of organisms displaying this feature. On one hand, some organisms live for an extended period of time without showing signs of aging. However, on the other hand, the longer they live, the higher is the probability of their death, which is usually preceded by more or less obvious deterioration of their morphology and fitness (delayed aging).

**Table 1:** An overview of different aging systems with the number of publications related to aging and longevity research according to the Entrez PubMed database as of January 20, 2020

System	Aging (ageing)	Longevity	Senescence
Mammals			
Human	284,999 (314,050)	24,826	2,98,989
* Homo sapiens*	271,352 (298,844)	23,005	2,84,079
Human fibroblasts	6465 (6495)	524	8595
Okinawa(ns)	178 (186)	99	188
Mouse	47,620 (49,934)	5648	52,502
* Mus musculus*	45,695 (47,865)	5428	50,284
* Rat*	50,502 (51,805)	2233	51,909
* Rattus norvegicus*	49,271 (50,487)	2148	50,569
Blind mole rats	19 (20)	17	19
* Spalax*	13 (13)	11	13
* Spalax judaei*	0 (0)	1	0
* Spalax golani*	0 (0)	2	0
* Spalax carmeli*	1 (1)	1	1
* Spalax ehrenbergi*	3 (3)	1	3
Naked mole rats	140 (143)	122	142
* Heterocephalus glaber*	61 (65)	55	64
Elephants	116 (116)	41	120
African elephants	81 (81)	28	81
* Loxodonta africana*	17 (19)	5	19
African elephants	28 (32)	6	31
* Elephas maximus*	75 (82)	29	82
Whales	140 (149)	53	152
Bowhead whales	16 (16)	11	16
* Balaena mysticetus*	14 (14)	9	14
Elephant seal	85 (87)	7	88
* Mirounga angustirostris*	4 (4)	1	4
* Mirounga leonina*	4 (4)	2	5
Bats	398 (437)	91	420
* Myotis brandtii*	3 (3)	3	3
* Myotis velifer*	2 (2)	3	2
* Desmodus rotundus*	2 (3)	4	2
* Myotis lucifugus*	7 (7)	3	7
* Rhinolophus ferrumequinum*	2 (2)	1	2
* Miniopterus schreibersii*	1 (1)	2	1
* Myotis myotis*	21 (21)	16	22
* Myotis bechsteinii*	1 (1)	1	1
Reptiles			
Turtles	110 (112)	45	114
Giant tortoise	4 (4)	4	4
Galápagos tortoise	4 (4)	3	5
* Chelonoidis nigra*	0 (0)	0	1
* Chrysemys picta*	9 (9)	5	10
* Emydoidea blandingii*	1 (1)	1	1
* Trachemys scripta elegans*	2 (2)	2	2
* Chelonoidis abingdonii*	1 (1)	1	1
* Aldabrachelys gigantea*	1 (1)	1	1
* Dermochelys coriacea*	2 (2)	1	2
* Caretta caretta*	11 (11)	1	14
* Chelydra serpentine*	0 (0)	0	0
* Pseudemys scripta*	1 (1)	0	1
Fishes			
Annual fish	131 (136)	69	135
* Nothobranchius*	106 (111)	56	108
* Nothobranchius furzeri*	69 (74)	38	71
* Nothobranchius guentheri*	22 (22)	10	22
* Nothobranchius rachovii*	8 (9)	5	8
Rockfish	11 (11)	11	11
Greenland shark	6 (6)	5	6
* Somniosus microcephalus*	2 (2)	2	2
Invertebrates			
* Caenorhabditis elegans*	3364 (3510)	2375	3415
* Drosophila melanogaster*	2902 (3014)	1830	2961
The ocean quahog	8 (8)	7	8
* Arctica islandica*	18 (18)	16	18
Jellyfish	20 (22)	9	22
* Turritopsis dohrnii*	3 (3)	1	3
* Turritopsis nutricula*	1 (1)	0	1
* Schmidtea mediterranea*	6 (9)	2	6
* Hydra*	53 (60)	22	61
* Hydra vulgaris*	5 (5)	1	5
* Hydra viridissima*	0 (0)	0	0
* Hydra oligactis*	7 (7)	2	8
Bacteria			
* Escherichia coli*	1161 (1260)	230	1278
* Caulobacter crescentus*	9 (10)	1	11
Fungi			
* Saccharomyces cerevisiae*	1755 (2035)	703	2047
* Neurospora spec*	66 (66)	16	101
* Podospora anserina*	137 (137)	48	179
* Aspergillus spec*	169 (169)	20	167
* Schizosaccharomyces pombe*	173 (173)	37	168
Protozoa			
* Paramecium caudatum*	8 (8)	2	10
3D cellular/acellular systems			
Organoids	173 (193)	6	195

The list of systems that we described in this article is our arbitrary decision. Nonetheless, our primary intention was to address systems representing a wide spectrum of phylogeny which, at the same time, display some unique traits and adaptations that differentiate them from other organisms, determining the particular course of their aging, longevity, or immortality. We believe that this study may attract the interest of readers to nonclassical biogerontological systems because of either the fascinating diversity of its countenances or the possibility that it will help interpret knowledge about aging from a wider, deeper, and, most of all, more critical perspective.

## Aging-like phenomena in prokaryotes

### Escherichia coli

The most common method of bacterial cell propagation is binary fission in which two genetically equivalent daughter cells arise [14]. Such reproduction is a source of dogma that, under permissive environmental conditions, bacterial cells are not subjected to any process that could be considered as reminiscent of aging. This simplistic view changed when symmetrically replicating *E. coli*—a Gram-negative bacillus belonging to the normal microbiota of mammals—appeared to inherit some functional features in a clearly asymmetrical manner. It has been found that two daughter *E. coli* cells, apparently identical, differ concerning some inherited intercellular elements due to uneven segregation of the maternal cell. This functional asymmetry means that part of the progeny received some preexisting constituents of the mother’s cells (‘old pole’), while the second cell produces these elements de novo (‘new pole’). As a result, the lineages that vertically inherited old poles developed features that could be considered aging, of which a slowed growth rate, reduced offspring, and increased plausibility of death were the most important. The reproductive capacity (lifespan) of this specific lineage was eventually terminated after approximately 100 divisions. Although the composition of the old pole is still poorly recognized, it seems probable that it contains fragments of the cell wall, modified DNA, and aberrant proteins [15].

The stationary phase of bacterial growth is another example of aging-like behavior, often called *conditional senescence*. This phase refers to an adaptive reaction when bacteria confront adverse environmental conditions, e.g., a lack of adequate nutrient supply. *E. coli* cells that enter the stationary phase display degenerative changes due to the accumulation of toxic metabolites, which leads to decreased proliferative potential [16]. Cells aged in the stationary phase display condensed chromosomes and translationally silent, dimerized ribosomes, which are attributed to Dps-related encapsulation of DNA [17] and guanosine pentaphosphate-related induction of ribosome modulation factor [18], respectively. Some changes have also been observed in their morphology: they become smaller and more spherical, which is linked with the activity of the sigma factor σ^S^/RpoS [19]. Relevantly, characteristics acquired by bacteria in the stationary phase, particularly growth reduction, are permanent, which means that they are preserved even when the cells are placed in a nutrient-rich environment [20].

As per the molecular mechanism of conditional senescence, cells with deteriorated growth are characterized by decreased expression of genes regulating carbohydrate catabolism and energy production (e.g., *sdhA* encoding succinate dehydrogenase; *cydA* and *cydB* associated with the subunits of cytochrome bd-I terminal oxidase) and macromolecule synthesis (e.g., *murI* that encodes glutamate racemase involved in peptidoglycan synthesis) [21]. Degenerative changes in starved bacteria may also be associated with the accumulation of aggregated, cross-linked, and unfolded proteins (e.g., DnaK/HSP70 chaperone and ClpXP protease) [22], providing a link between bacterial aging and the loss of proteostasis. Research using individual-based systeming showed that the transfer of protein aggregates into old poles proceeds via passive diffusion and that the structures are primarily displaced to regions corresponding to the nucleoid-free space in the pole, pointing to the significance of increased macromolecular crowding in the nucleoids [23]. Other events associated with the polar transfer of proteins have been described in the elegant review by Laloux and Jacobs-Wagner [24].

Aged *E. coli* cultures accumulate carbonylated proteins, which is suggestive of the involvement of oxidative stress [25]. This prediction was in line with the observation that *E. coli* defective in genes coding for the antioxidative enzymes superoxide dismutase (SOD) and catalase (CAT) displayed a high content of oxidized proteins upon starvation [26]. The accumulation of aberrant proteins is not a direct effect of reactive oxygen species (ROS) activity, the production of which remains unchanged [27], but rather a result of the specific vulnerability of some proteins in the stationary growth phase to oxidative modifications, likely due to defects arising during translation, e.g., framing and missense mistakes and stop codon read-through [28]. The magnitude of proteostasis disruption is also determined by the dosage of external stressors, which allows for the maintenance of equilibrium between cell aging and immortality. Upon cell exposure to a high level of stress, the damage to proteins exceeds some threshold, and the equilibrium shifts towards aging and mortality [29]. Of note, the accumulation of abnormal proteins that can restrict cell reproductivity has been found in old poles of bacteria maintained in optimal growth conditions [30], which implies that impaired proteostasis may be the core event in *E. coli* aging, irrespective of its trigger.

Last but not least, the emergence of protein aggregates and their asymmetric partitioning is linked by some authors with favorable effects for bacterial cells. According to Govers et al., aggregates accumulate in response to sublethal proteotoxic stressors rather than in response to the aging-related decline in proteostasis, and their inheritance and wide distribution within the next generations of bacteria allow them to cope with aggregate-inducing stressors far more effectively than their ancestors [31].

### *Caulobacter crescentus*

**Fig. 1:**
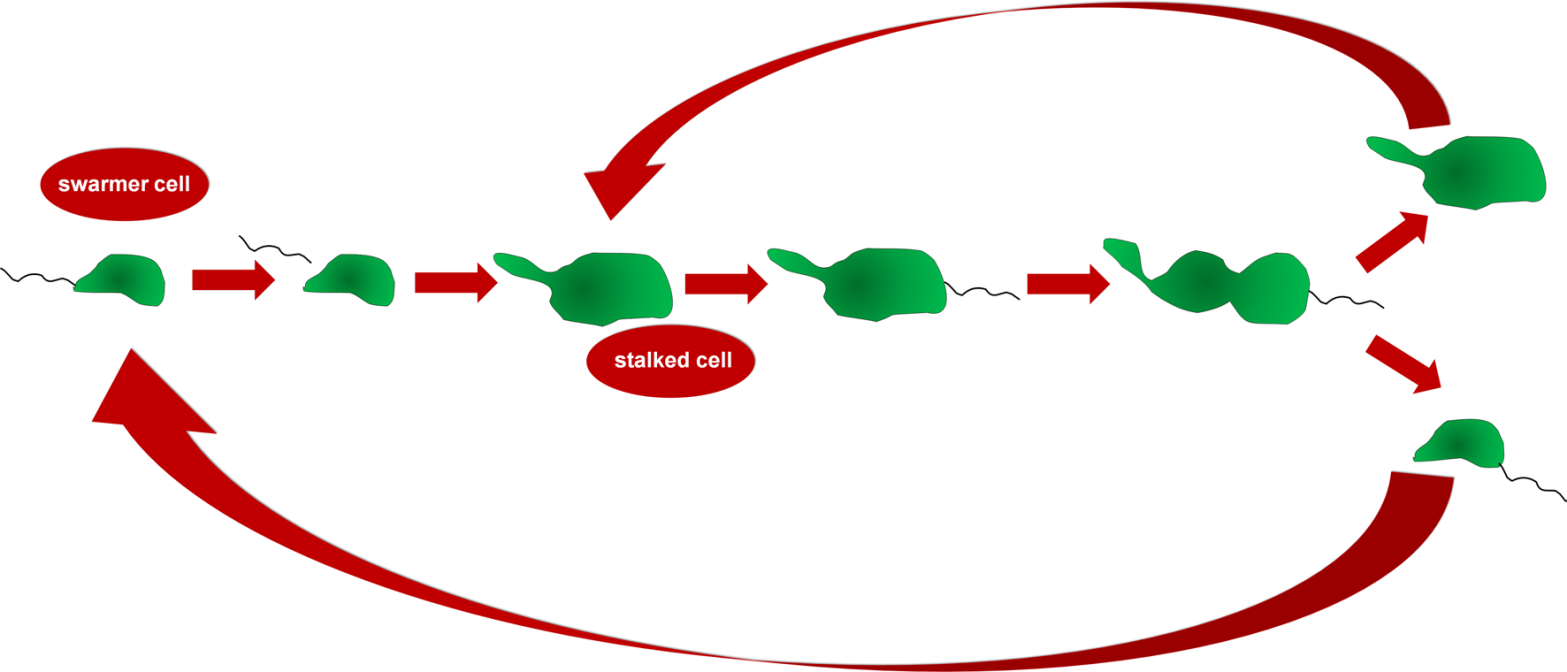
The asymmetric division and the unique dimorphic life cycle in Caulobacter crescentus

*Caulobacter crescentus* is a Gram-negative α‐proteobacterium living in freshwater habitats. Because freshwater environments display relatively high variations in food availability and microbial composition, their residents are known to very flexible in terms of their adaptations that allow them to survive. *C. crescentus* is also known to be capable of changing its morphology in response to external factors. Namely, when the bacterium experiences damage to the cell membrane, it blocks divisions and develops filamentous appearance [32]. Another unique feature of *C. crescentus* is an exceptional dimorphic life cycle and asymmetric propagation pattern which also can be treated as unique aptitudes to variable living conditions. The dividing parental *C. crescentus* cell delivers two categories of offspring cells, distinct in terms of both structure and function (Fig. 1). A mobile swarmer cell equipped with a flagellum arises first. Upon some minutes of swimming, the cell loses the flagellum and converts into a sessile form in which a stalked tubule-like structure salient from one end permits the cell to attach to surfaces with a polar staple. Of these two daughters, only the stalked cell replicates DNA and goes to the next round of the division cycle [33]. The time a stalked progeny needs to form the subsequent swarmer cell steadily increases, which has been considered a sign of aging [34].

From a mechanistic point of view, the molecular machinery of aging in *C. crescentus* is elusive. What is known is that aged cells may accumulate aggregated proteins which may suggest that defective proteostasis plays some role in this process. The aggregates form as multiple distributed foci located throughout the cell volume; however, under mild stress conditions, the majority of these structures are efficiently dissolved by the chaperone DnaK and the disaggregase ClpB. Persistent aggregates accumulate when the magnitude of stress increases or when the cells are subjected to genetic deterioration of the protein quality management machinery. These aggregates are not allocated to the cell poles or transferred to only one progeny type, as occurs in *E. coli* cells; conversely, they are deployed proportionally to both swarmer and stalked forms [35].

*Caulobacter crescentus* may also be used in studies on the evolutionary basis of aging, which revealed experiments by Ackermann and colleagues [36]. They designed bacterial populations undergoing intense selective pressure early in life and weak late in life in which the manipulation resulted in a markedly decreased population doubling time after several generations. This behavior is typical for the selective removal of a beneficial mutation in populations of microorganisms reproducing asexually [37] and probably occurs due to mutations that emerged in the tested organisms. As per a phenomenology of aging itself, some populations evolved slower aging compared with original cells, which was evidenced by a slower decline in reproducibility with age. At the same time, some of the clones varied in the rate of aging, which displayed a slower or faster pace of this process than their progenitor. The second scenario, which was more frequent, indicates that at least one mutation that triggered faster aging had to accumulate to confer some benefits early in life. This, in turn, stays in line with the theory of antagonistic pleiotropy, assuming that there is an evolutionary trade-off mechanism between early life fitness and late-life mortality [38].

## Mechanisms to combat aging and death in hydrozoans

**Fig. 2:**
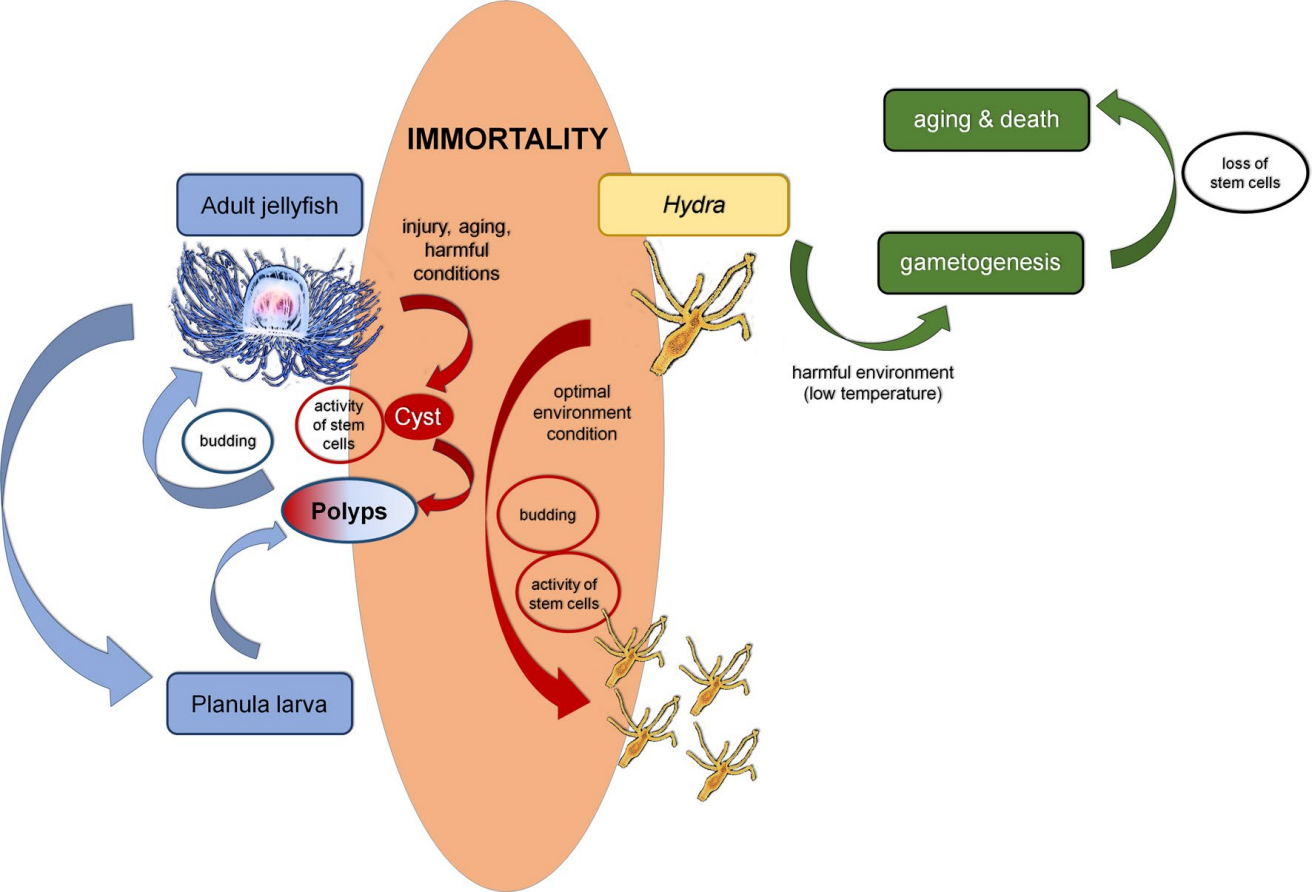
Mechanisms of immortality in *Turritopsis dohrnii* (aka Jellyfish) and *Hydra vulgaris*. In both cases, asexual reproduction provides new intact animals, although in Jellyfish, the immortality program is launched to fight against harmful environmental conditions, and in Hydra, it is an innate element of its biology. Conversely, in *Hydra oligactis,* unfavorable conditions (e.g., decreased temperature) activate sexual reproduction, which leads to the loss of budding ability, decreased activity of stem cells, aging, and death

Hydrozoans are a group of invertebrates within the phylum Cnidaria. They have a primitive nervous system and display the greatest plasticity with respect to morpho-genetic abilities, ecosystems, and behaviors in response to environmental factors among cnidarians. They also have a unique life cycle pattern in which an adult medusa produces tiny, free-swimming, and short-lived planula larvae. Upon the planula settling down on the seafloor, it transforms into a modular colony of polyps that reproduce asexually delivering new medusae by budding. Mostly, the medusae proceed through the phase of sexual maturity and one or a few cycles of reproduction (gamete production), which culminates in their gradual disintegration and death [39]. A representative of hydrozoans, *Turritopsis dohrnii*, exhibits an exceptional ability to avoid death: when facing an injury, aging or unfavorable environment, its medusal form shrinks, loses its swimming skills, and undergoes a retrograde transformation to a chitin covered, poorly differentiated cyst, which eventually gives rise to a preceding juvenile morph, the polyp [40]. This specific metamorphosis, in a direction opposite to the typical ontogenetic path, caused *T. dohrnii* to gain a colloquial name—the immortal jellyfish (Fig. 2) [41].

The observation that the medusa and polyp differ in terms of somatic cells in the umbrella (apart from obvious differences in shape and anatomy) allowed Piraino et al. to use a selective excision procedure to evaluate a mechanism of the reverse transformation and functional rejuvenation of *T. dohrnii.* The study revealed that the process requires differentiated cells of the exumbrellar epidermis and portion of the gastrovascular structures, and is based on their transdifferentiation into perisarc-producing cells of the external envelope. The authors also consider that some role may also be played by interstitial stem cells (so-called I cells), which, despite their vigorous proliferation and ability to differentiate into other cell types, do not contribute to the development of the chitinous coat [42]. Further research employing transcriptomics showed the plausible driving forces underlying the reverse life cycle in *T. dohrnii*. Namely, they showed that a cyst, as the intermediate form in the life of *T. dohrnii*, between the morphs of the medusae and the polyp overexpresses transcripts coding for DNA synthesis, integration, and telomere maintenance, whereas transcripts associated with the mitotic cycle, aging, and protein synthesis were underexpressed [43]. Taking into account the unique way that *T. dohrnii* avoids death and lives, at least theoretically forever, this organism seems to be an excellent tool for detailed examination of the factors governing aging and immortality. Knowledge about the molecular biology of this organism is, however, very limited. Recently, its mitochondrial genome has been sequenced [44]; albeit there is still a lack of data regarding its whole genome sequence.

## Hydra and its (non)aging strategy

*Hydra*, a tiny cnidarian polyp and the superior cousin to the immortal jellyfish, is known to have an extraordinary capacity for self-renewal throughout its lifetime and negligible aging [45]. The animals have a tubular body with the head at the apical extremity and the foot localized at the other extremity. The head is comprised of a dome called hypostome which is terminated by the mouth opening at its tip and a flange with tentacles at its bottom. Robust regeneration of *Hydra’s* body occurs after an injury to an animal, e.g., experimental bisection [46]. A complete polyp may also arise from clusters generated from aggregating cells upon their preceding dissociation [47]. Remarkably, the regeneration of *Hydra* does not involve cell proliferation, which was found, e.g., in experiments showing that the polyp’s head was rebuilt from endodermal epithelial cells expressing a fluorescence tracker and proceeds in the absence of local cell divisions [48]. Such regeneration is called morphallaxis and means that the rebuilt process is based primarily on tissue patterning and reorganization of previously existing structures. Such a mechanism of regeneration differs from epimorphosis in which active proliferation plays a major role [49].

Under optimal, nutrient-rich conditions, *Hydra* replicates asexually by budding, and thus, new populations of stem cell-derived cells forming the buds determine the long-lasting life of these animals and biological immortality. Seminal observations in this regard pointed to the lack of aging in polyps of *H. vulgaris*, *H. viridissima*, and *H. oligactis* [50]*.* Jones et al. estimated that the lifespan of *H. vulgaris* in laboratory conditions would be approximately 1400 years, with steady rates of fecundity and mortality (Fig. 2) [51].

It is believed that *Hydra* polyps may avoid aging thanks to the vigorous activity of their three separate populations of stem cells [51], which are unipotent endodermal and ectodermal epithelial cells and multipotent interstitial cells [52]. These cells self-replenish along the body column and then differentiate, providing various populations of cells that are displaced towards the apical edgings (foot and tentacle regions) of the animal [53]. It has been estimated that all cells within *Hydra’s* body are replaced approximately every 20 days [54]. Recently, single-cell RNA sequencing was used to identify the molecular fingerprints of stem cells and differentiated cells in *Hydra*, and to delineate the differentiation trajectories and related transcription factors for each cell lineage [55].

Particular attention concerning *Hydra* transcription factors has been paid to forkhead box O (FoxO), which is known to play multiple roles in vital cellular processes, such as apoptosis, proliferation, autophagy, differentiation, immunity, and resistance to oxidative stress [56]. Boehm et al. showed that the explicit ability of *Hydra* to self-renew may be associated with the expression of FoxO, which positively regulates the maintenance of the proliferative potential of interstitial stem cells and progenitor cells. When the regeneration capabilities were abolished by targeting FoxO, the number of terminally differentiated somatic cells increased at the cost of a decline in the growth rate of the population [57].

Although the permanent proliferation of stem cells generates damage to cellular structures, this damage does not prevent *Hydra’s* immortality. It has been proposed that this damage resistance results from a high proportion of mitotically active cells relative to their nondividing counterparts within each polyp and the continuous replacement of cells that accumulate damage by plentiful stocks of constantly self-renewing stem cells, their differentiation, and/or elimination by programmed death or bud production [58]. The high proportion of stem cells and their robust replication also allow adaptation of the non-aging phenotype in *H. vulgaris* despite the presence of a wide range of abnormalities in lamin protein and the nuclear envelope composition [59]. A transfer of error-free DNA to the next generations of stem cells is possible thanks to effective DNA repair mechanisms. Experiments on *H. vulgaris* showed the expression of nucleotide excision repair pathway homologs, including *XPA* and *XPF* [60].

An opposite behavior to non-aging *H. vulgaris* has been found in the case of another *Hydra* representative*, H. oligactis*. Upon transfer of these animals from 18 to 10 °C, the polyp undergoes low temperature-dependent gametogenesis, plausibly initiated by the loss of interstitial stem cells [61]. Moreover, it loses the ability to bud, which eventually leads to accelerated aging followed by high mortality (Fig. 2) [50]. The remaining features of sexually differentiated *H. oligactis* aging include a decreased capacity for food capture, reduced spontaneous contractile movements (due to incompetence of the actin fibers), and deteriorated reproduction [62]. The development of an aging phenotype in sexually reproducing *Hydras* has also been described in *H. canadensis* and *H. oxycnida* [63]. Interestingly, very poor propagation in culture conditions and negligible egg production are not only signs of *H. oligactis* aging. Similar behavior has been found in polyps bearing spontaneously developed tumors that originated by the differentiation arrest of female gametes [64].

Some significance for the understanding of cold-driven aging in *H. oligactis* may have been observed by Bosch et al., who demonstrated that these animals are highly thermosensitive. Conversely, for *H. vulgaris,* which was able to survive exposure to high temperature (33 °C) for up to 90 min, *H. oligactis* polyps maintained under the same conditions for up to 1 h degenerated and died. The thermotolerance of *H. vulgaris* was attributed to its ability to synthesize heat-shock protein (60 kDa in size), whereas *H. oligactis* failed to produce any detectable temperature-reactive proteins in response to heat [65]. In line with these observations, another report showed that a lack of stress reaction in *H. oligactis* might be associated with its low ability to synthesize heat-shock protein 70 mRNA [66] and its reduced stability [67].

Direct comparison of *H. oligactis* epithelial stem cells in cold-sensitive (Ho-CS) and cold-resistant (Ho-CR) organisms showed that the self-renewal ability of these cells in aged Ho-CS animals is permanently decreased, whereas in non-aging Ho-CR individuals, it is preserved. Further research showed that the development of the aging phenotype in some hydras might depend on the efficiency of autophagy. This process in Ho-CS epithelial cells was found to be defective, as evidenced by the presence of deficient autophagosome development. Moreover, these cells accumulated the autophagosome cargo protein p62/SQSTM1, demonstrating an inappropriate reaction to starvation and low efficiency of autophagy induction after neutralization of the proteasome. When autophagy was inhibited by knocking down WIPI2, the aging phenotype was inducible in Ho-CR animals [68]. Another feature of Ho-CS *H. oligactis* is a progressive loss of neurogenesis, which is another harbinger of their inevitable aging and death. Mechanistically, this deterioration of the neural system may be associated with the downregulation of two proneurogenic agents, the homeoprotein prdl-a and the neuropeptide Hym-355 [69].

Animals sensitive to aging in which sexual reproduction is induced and those avoiding this aging due to asexual breeding differ concerning the presence of gametes. The trade-off between reproduction (−) and aging (+) in *H. oligactis* that is reflected by increased generation by interstitial stem cells of gamete precursors at the expense of interstitial stem cell production is in line with Kirkwood’s disposable soma theory of aging, according to which aging and mortality of the soma are the prices an organism must pay for its fertility [70]. On the other hand, the lack of an apparent distinction between germ and soma cells (interstitial stem cells are the source of both germline and somatic cells [71]) combined with negligible aging and the constant rates of age-dependent death and reproduction in *H. vulgaris* [72] challenges the postulated universality of the major evolutionary theories of aging [73]. Nonetheless, despite this dichotomy in aging behavior in different representatives of *Hydra*, this animal constitutes an excellent tool for research on molecular, cellular, and environmental factors determining the transition between aging and immortality. Importantly, however, most of the research on *Hydra* to date was mainly focused on its exceptional regeneration, which means that several important informations related to its aging-free biology may still be obtained. In this context, the lack of the longstanding observations and analyses of individual organisms from hatching and then over very long period of time (decades?) seems to be the most severe [74].

## The ocean quahog

The ocean quahog (*Arctica islandica*) is the North Atlantic Ocean native bivalve mollusk, found burrowed in the top layer of sand and muddy substrates at a water depth between 25 and 80 m [75]. It matures very slowly, because the average age of sexual maturity for Nova Scotian animals is 13.1 and 12.5 years for males and females, respectively [76]. At the same time, *A. islandica* is the longest-lived non-colonial organism known to science so far. An analysis of the annual growth bands on the surface of the outer shell margin revealed that Hafrún, a clam native to the northern coast of Iceland, may live as much as 507 years [77].

Research by Abele et al. showed that the long lifespan of this marine invertebrate may result from a low magnitude of oxidative stress. Namely, they found that *A. islandica* displays stable antioxidative protection provided by various systems, including CAT, citrate synthase activity, and glutathione [78]. This seems to result in the lower production of reactive oxygen species (hydrogen peroxide) and decreased content of oxidation products (carbonylated proteins) in *A. islandica* tissues compared with the shorter lived clam *Mercenaria mercenaria* [79]. It is likely that relatively low oxidative stress in *A. islandica* may be, at least partly, associated with some adjustments in metabolism to self-induced hypoxia and metabolic rate depression during burrowing [80]. In fact, no ROS burst was found in isolated tissues during the hypoxia/reoxygenation state, and antioxidant enzyme (SOD, CAT) activities were not elevated in metabolically suppressed animals compared with their normal breathing counterparts [81]. This may suggest that these animals may lower their lifetime oxidative stress by interval entry into energy-saving behaviors. Significantly, periods of hypoxia do not yield increased amounts of anaerobic metabolites, such as octopine, lactate, and succinate, which implies that *A. islandica* maintains aerobic biochemistry even under low levels of environmental oxygen [82]. Some adaptations which may also explain the longevity of *A. islandica* are also associated with some specific features of mitochondrial metabolism, including increased resistance of mitochondrial membranes to peroxidation [83] and low hydrogen peroxide production linked to complexes I and III activities [84]. These features do not correlate, however, with longevity across different populations of *A. islandica* [85].

The above-mentioned findings are consistent with observations by Gruber et al., who compared long-lived (Icelandic animals) and short-lived (Baltic Sea animals) representatives of *A. islandica* and found that both population express well-preserved cellular maintenance systems, as evidenced according to the lack of changes in protein and lipid oxidation with age. The magnitude of nucleic acid oxidation was the only parameter which displayed the age-related increase and the level of damage in short-lived organisms and dynamics of the damage accumulation were higher than in long-lived animals. The latter were, in turn, characterized by higher resistance of their proteins to unfolding stress caused by the treatment with urea, which may imply the role of well-preserved proteostasis as one of plausible determinants of *A. islandica* longevity [86]. This assumption agrees with the observation by Treaster et al., who found no increase in *A. islandica* global proteome unfolding in response to several stressors and linked this effect with activity of small molecular chaperones [87]. Other report shows no significant relationship between the extent of protein ubiquitination and age of these organisms [88]. To some extent, these findings contrast, however, with research by Ungvari et al., who failed to demonstrate the augmented protein recycling in long-lived *A. islandica* than in short-lived *M. mercenaria* [79]*.*

Another explanation of differences in lifespan within *A. islandica* populations may be varied level of environmental insult experienced by Icelandic animals and their counterparts living in the Baltic Sea. In brief, the short-lived *A. islandica* may experience more stress resulting from fluctuations in temperature, oxygen availability, and salinity, whereas the long-lived animals live in more stable conditions [89]. At the same time, it must be stressed that, although the both discussed populations of *A. islandica* remarkably differ with respect to their maximum lifespan (226 vs. 36 years), genetic tests classify them into the same species [90].

As per another indicator of aging, that is the length and shortening of telomeric DNA, a comparative study in which telomeres and telomerase were compared in short-lived and long-lived *A. islandica* showed that both organisms display high heterogeneity in telomere length and constant telomerase activity, irrespective of animal age. Because telomere length was stable, it would be considered one of the cellular mechanisms responsible for the long lifespan of these metazoans [91]. A lack of telomere disruption may also result from the high resistance of *A. islandica* to genotoxic insult generated, e.g., by exposure to a robust exogenous oxidant, tert-butyl hydroperoxide (t-BHP). Experiments showed that the survival of *A. islandica* exposed to t-BHP was markedly longer than that of *M. mercenaria,* which could be, at least partly, explained by the higher resistance of these organisms to t-BHP-induced apoptosis [79].

## Aging in the fish of the genus *Nothobranchius*

Annual fish of the genus *Nothobranchius* are used as a system of aging owing to their naturally short lifespan (median survival ranging from 9 weeks in *N. furzeri* to 12 months in *N. guentheri*) and nonoverlapping generations. These organisms display sexual diversity and procreate by producing eggs that are resistant to draining and even require a dry period to develop accurately [92]. A vital element of the survival of eggs is their ability to enter diapause [93]. Aging of the genus *Nothobranchius* representatives is associated with a reduction in locomotor functions [94] and degenerative changes in morphology, including slimming and thinning. Male specimens lose their unique bright coloring, whereas females exhibit a body deformation from rotund-like shape to curved spine appearance [94]. Aged *Nothobranchius* species display degenerative lesions accumulating in the liver, kidney, heart, and gonads and an increased frequency of neoplasms [95]. As per reproductive aging, the reports provide conflicting results. According to some, aged representatives of *Nothobranchius* display a decline in fertility and fecundity [92], whereas others indicate that there is no detectable drop in absolute female egg delivery, although relative fecundity (egg production controlled for female body mass) tends to decline along with the severe deterioration of the gonads [96].

*Nothobranchius furzeri* is the shortest-lived vertebrate that can be farmed in captivity [97]. In terms of aging, this fish shows several similarities with normal somatic cells of human origin. Aged animals display increased activity of senescence-associated β-galactosidase (SA-β-Gal)—the universal marker of cellular senescence [98]—in dermal fibroblasts and accumulate the product of lipid peroxidation, lipofuscin [92]. The accumulation of lipofuscin in short-lived aged strains of *N. furzeri* appeared to be higher than that in age-matched long-lived animals, which was accompanied by a higher magnitude of cognitive deterioration [99]. The role of oxidative stress in *N. furzeri* aging revealed observations by Milinkovitch et al. [100], who demonstrated that the magnitude of lipid peroxidation, determined according to the production of malondialdehyde (MDA) in the liver and muscles of 30-week-old organisms, is higher than that in their 18-week-old counterparts.

Unexpectedly, the MDA level in the liver of middle-aged organisms was lower than that in 7-week-old juveniles, which may reflect the high metabolic demands of the liver and concomitant overproduction of ROS associated with an explosive phase of early development in the organism [101]. The age-associated exacerbation of oxidative stress has also been reported in other representatives of *Nothobranchius*, including *N. rachovii* [102] and *N. guentheri* [103]. In the latter, aging was also associated with decreased activity of the antioxidative enzymes SOD, CAT, and glutathione peroxidase (GPx) [103].

The accumulation of lipid peroxidation products seems to depend on the temperature to which the fish is acclimatized. The available studies, however, provide contradicting findings. In the above-cited study by Milinkovitch et al., the concentration of MDA in the liver of aged animals was higher in fishes maintained under suboptimal 22 °C than under 26 °C. In the muscles, the MDA level did not depend on temperature [100]. The former observation contrasts with the study by Valenzano et al., who measured the lipofuscin level in the liver cells of aged *N. furzeri* and found that it accumulates less efficiently at 22 °C than at 25 °C [104]. The differences between the above-mentioned reports may stem from the differences in farming and lifetimes of the strains used for experiments. In the latter report, decreasing temperature increases either the median or maximum lifespan of *N. furzeri,* which proceeds with a simultaneous upgrade of locomotor and learning functions.

These findings are consistent with a report by Lu and Hsu, who found that a reduction in ambient temperature extends the lifespan of *N. rachovii.* Mechanistically, this effect was probably linked with an improvement in cellular degradation pathways, as evidenced by increased 20S proteasome activity, decreased levels of polyubiquitin aggregates, and increased expression of the macroautophagy indicator microtubule-associated protein 1 light chain 3 (LC3) [105]. Another intriguing factor affecting the lifespan of *N. furzeri* appeared to be the gut microbiota. This conclusion originates from the observation that the transplantation of gut bacteria from young donors into middle-aged individuals led to an extension of their lifespan and a reduction in behavioral decline [106]. Of note, the four most common bacterial phyla present in *N. furzeri* intestines, i.e., Actinobacteria, Bacteroidetes, Firmicutes, and Proteobacteria, are the same as in humans [107]. The lifespan of *Nothobranchius* populations may also depend on the humidity of their environment. Namely, populations originating from dry regions were characterized by a shortened lifespan in captivity compared with their counterparts from humid regions [108]. Shortened lifespan and accelerated development of the senescence phenotype also describe inbred laboratory strains of *N. furzeri* compared with wild-derived organisms [99]. The group of Valenzano also revealed using gene sequencing analysis that short-lived annual fishes have larger genome than their non-annual counterparts due to the accumulation of several repetitive elements, allowing the accumulation of deleterious mutations. Several of these mutated genes are known to act as the lifespan regulators and include *mtor*, *insr*, *ampk*, *foxo3*, and *polg*. At the same time, the annual fishes exhibit some beneficial mutations in genes associated with development and reproduction, which was indicative for their positive selection. These results suggest that short-lived animals, especially those having limited genetic diversity, may possess a lot of mutations that escaped the selective pressure and finally became widely distributed limiting their lifespan [109].

From a molecular perspective, aged *N. furzeri* display downregulated activity of histone deacetylase class I (HDAC) orthologs, which are critical for epigenetic regulation of chromatin structure and are linked with the development of aging and certain age-dependent diseases [110], in muscles, liver, and brain [111]. A similar age-dependent decline was also reported in the case of whole fish mRNA analysis for class III HDACs (sirtuins), including *sirt1*, *sirt2*, *sirt5a*, and *sirt7*. In the spatiotemporal context, the transcriptional activity of *sirt1*, *sirt5a*, *sirt5b*, *sirt6*, and *sirt7* was decreased in muscles and intestines, but at the same time, mRNA levels for *sirt2*, *sirt3*, and *sirt4* exhibited an age-dependent increase in intestines with concomitant downregulation of sirt2 mRNA levels in muscles [112]*.*

Tissue-specific changes in sirtuin mRNA seem to be connected with some alterations observed, e.g., in mitochondrial metabolism. Hartmann et al. have shown that mitochondrial DNA copy number decreases during *N. furzeri* aging. This alteration is accompanied by reduced activity of PGC-1α, an enzyme engaged in mitochondrial biogenesis, in muscles, which coincided with decreased activity of mitochondrial oxidative phosphorylation system (OXPHOS) elements, particularly complexes III and IV, and deteriorated ADP-stimulated and succinate-dependent respiration, pointing to the role of mitochondrial dysfunction in *N. furzeri* aging [113]. Of note, sirt5a has been found to act as protein lysine demalonylase and desuccinylase [114], which may indicate that decreased transcription of this HDAC may somehow contribute to aberrant mitochondrial function in aged *N. furzeri* muscles.

The downregulated brain activity of class I HDACs is correlated with enhanced expression of transcripts for the effector of telomere-dependent senescence in somatic cells [115] and the cell-cycle inhibitor p21 [111]. The increased skin expression of p21 and Cdkn2a/b (homologs to human p16 and p15) with age was evident in long-lived strains of *N. furzeri* but not in their short-lived counterparts, which may imply that changes in cell-cycle inhibitors may be linked with chronological aging rather than with a biological process. More importantly, when primary cells were established from *N. furzeri* skin and fins and allowed to proliferate for an extended time, they failed to adopt classic signs of replicative senescence, including growth arrest in G_1_ phase of the cell cycle, altered morphology, increased doubling time, and elevation of p21 and γH2AFX levels, irrespective of the strain’s lifespan [116]. The authors of the study link this lack of apparent senescence phenotype with high expression of telomerase and unaltered telomeres; however, they do not take into account that cellular senescence may proceed via telomere-independent machinery [117].

The role of telomeres in the senescence of *N. furzeri* cells seems to still be unclear, as it has been reported that the aging of long-lived strains of *N. furzeri* is associated with significant telomere shortening in muscles and skin, while short-lived individuals age without erosion of telomeric DNA. Unexpectedly, tissues of the long-lived strains were also characterized by an up-regulated TERT subunit of telomerase, which appeared to be unable to prevent the age-related shortening of telomeres [118]. This may suggest that DNA damage responsible for DNA shortening exceeded the repair capacity of telomerase or that telomerase exerts some alternative, telomere length-independent functions in *N. furzeri* [119]. In *N. guentheri*, in turn, there was no change in telomerase activity with age [103]. It should be emphasized that unpredictable interactions sometimes occur along the lines of the telomerase-telomere relationship in human cells. This is the case, e.g., in normal peritoneal mesothelial cells in which cellular senescence is accompanied by increased telomere length in the presence of decreased expression of hTERT [120].

As per SA-β-Gal, which is abundant in aged individuals [92], primary *N. furzeri-*derived cells display detectable and stable levels of this enzyme activity throughout their whole replicative history, which supports the conclusions of some other groups who postulate that in some experimental systems, e.g., in neurons, SA-β-Gal activity is not a universal and solid marker of cellular senescence [121]. The results presented above also suggest that all observed age-associated changes in *N. furzeri* are mechanistically separated (independent) from cellular, proliferation-related phenomena, such as cellular senescence.

## The mystery of Greenland shark longevity

Radiocarbon tissue analysis showed that Greenland sharks (*Somniosus microcephalus*) belong to the longest-living vertebrates, as their lifespan may reach at least 392 ± 120 years [122]. Comparative analysis with other species showed that these sharks display high values of GPx in muscles and low levels of carbonylated proteins in erythrocytes, suggesting a beneficial proportion of redox parameters. At the same time, oxidative status was not correlated with longevity, which is instead a form of adaptation to specific environmental conditions [123]. Despite the fascinating value of the Greenland shark’s lifespan, neither cellular nor molecular determinants of this phenomenon have been identified. It is plausible that very long lifespan of these animals may be associated with certain environmental reasons, particularly minimal predation [73].

## The extraordinary lifespan of naked mole rats

Naked mole rats (*Heterocephalus glaber*; NMRs), native to East African deserts, are the longest-living rodents known. These mouse-sized animals live in subterranean burrows, where they form breeding colonies with strictly defined social hierarchy and behaviors [124]. Observations by Buffenstein of the animals kept in captivity indicate that NMRs have an extraordinarily long lifespan, as they can live more than 30 years [125]. This value is five times longer than allometric predictions based on body size (~ 40 g) [124]. Between 2 and 24 years, NMRs display constant body composition and a lack of age-associated decline in their appearance and physiological functions typical for other mammals. Aged individuals do not display deteriorated cardiovascular function (e.g., sustained left-ventricular activity, lack of cardiac hypertrophy or arterial stiffening [126], and nitric oxide-dependent relaxation [127]), muscle structure or function (fiber integrity, mitochondrial ultrastructure) [128], bone quality [129], or cognitive functions [124]. At the same time, they do exhibit some typical age-associated changes seen in other mammals, such as an accumulation of lipofuscin in various organs, infarcts in the liver and kidneys, and retina degeneration [124]. Remarkably, female individuals show no deterioration of reproductive functions even at the end of their lifetime [130].

A detailed analysis of a collection of historical NMR lifespan data based on more than three thousand data points with Kaplan–Meier analyses showed that unlike other mammals, there is no apparent increase in the age-dependent mortality rate (Gompertz-Makeham law of mortality [131]), which led to the conclusion that NMRs represent a non-aging organism [132]. It is rational that the extremely long lifespan of NMRs may be associated, at least to some extent, with their strictly underground lifestyle that minimizes their risk of external causes of death, such as predation or hazardous atmospheric factors [133]. Primary sources of exceptional longevity in these animals are, however, supposed to originate from their unique physiology and evolutionary adaptations.

One of the most striking features of NMR biology is their tolerance of putatively hypoxic conditions characterizing their predominantly underground habitat. Unlike the majority of mammals whose tolerance of hypoxia is very low [134], NMRs evolved substantial metabolic adaptations to live and thrive in low oxygen pressure. Even at 3% oxygen, NMRs remain active and warm and are still able to explore their burrows [135]. Adaptative traits in NMRs experiencing hypoxia involve metabolic rate suppression, which is followed by a drop in body temperature, behavioral activity, and breathing and heart rates. Organismal energetics is switched towards intensified glycolytic metabolism, supported by the increased mobilization of liver glucose, which is surprisingly not accompanied by the development of metabolic acidosis. Depressed metabolic rates and remaining physiological functions that were diminished by hypoxia return to prehypoxia baseline levels during reoxygenation [136]. At the same time, NMRs maintained under low oxygen display an overexpressed (vs. hypoxia-sensitive mice) transcription factor HIF-1α and its target, vascular endothelial growth factor (VEGF) [137], whose effects could evolve to promote improved delivery of oxygen through increased permeability of existing blood vessels [138].

Paradoxically, although NMRs spend the majority of their lives in low oxygen conditions, the magnitude of oxidative stress in these organisms is relatively high. Comparative analysis of young and healthy NMRs with physiologically age-matched mice showed that the former generate more mitochondrial and cytosolic ROS [139]; accumulate more damage to DNA (2–8 times), lipids (2 times), and proteins (1.5–2 times); and have a decreased level of the antioxidant-reduced glutathione [140]. Interestingly, the activity of GPx in NMRs was also several times lower than that in mice, but the activities of superoxide dismutase, catalase [141], and α-tocopherol [142] were higher, plausibly contributing to the maintenance of some equilibrium between pro- and antioxidative processes. All these observations suggesting a strong oxidative insult in NMRs living in hypoxia are generally in keeping with the study by Magalhães et al., who showed that hypoxia elevates the level of protein carbonyl groups (read: oxidative stress) in mouse skeletal muscles, despite decreased mitochondrial respiration [143]. From a mechanistic point of view, the increased generation of oxidants in hypoxia is possible when there is either a high reductive capacity of a system (e.g., high NADH-to-NAD^+^ ratio) or a sufficient level of oxygen available for a reaction [144].

With regard to the age-associated changes in NMR biology, the animals appear to be resistant to oxidative stress during aging, as old individuals display unaltered levels of lipid peroxidation compared with their young counterparts [145]. This may result from the lack of changes in superoxide radical anion and hydrogen peroxide production during NMR aging, which contrasts these organisms with aging rats in whom both types of ROS were significantly overproduced [127]. Remarkably, the arteries of NMRs appeared to be highly insensitive to the proapoptotic effects of ROS, while mouse arteries were not [139]. A plateaued level of ROS is accompanied by a stable level of the antioxidative enzyme activities of SOD, CAT, and GPx, which contrasts NMRs from mice in which CAT and GPx activities declined with age, whereas the activity of manganese SOD was increased [141].

The lack of oxidative stress exacerbation during NMR aging may be partly guaranteed by an unaltered expression of complex IV mitochondrial enzyme and even decreased expression of complex I [128]. The maintenance of mitochondrial function during NMR aging corresponds to a lack of altered levels of genes coding for mitochondrial proteins, such as *NDUFB11*, *ATP5G3*, and *UQCRQ* [146]*.* It cannot be ruled out that long-term (from the juvenile stage) and stable oxidative stress could lead to some hermetic-like adaptations in NMRs in which an insulting agent provokes mobilization of various prosurviving mechanisms [147], analogous to the life-prolonging effects of caloric restriction [148].

Taken together, these findings indicate that neither the magnitude of oxidative stress nor protection against oxidants from the side of antioxidative systems seem to be decisive for the extreme longevity of NMRs. This indicates, in turn, that the free radical theory of aging clearly fails in the case of these animals and that late aging may result from other metabolic traits, such as proteostasis or cellular senescence patterns.

As per protein quality control, when young NMRs were compared to age-matched mice, they appeared to have much higher proteasome activity, which was accompanied by a lower degree of protein ubiquitination. Moreover, in contrast to mice that showed a significant age-dependent augmentation of the oxidation of cysteine residues, a measure of thiol group oxidation in proteins, and increased levels of ubiquitination, none of these effects were present in NMRs [149]. These findings suggest that proteostasis may be the key element responsible for longevity in NMRs and other long-living species [150]. Of note, in humans, there is an age-related weakening of proteasome activity, which is considered a cause of the accumulation of abnormal, aggregated, misfolded, and/or cross-linked proteins known to be linked with the development of certain cellular and systemic abnormalities in elderly individuals [151]. Another elements of quality control whose activity may matter with respect to the long life span of NMRs may be effective DNA damage repair and cell death of damaged cells. NMRs display up-regulated several genes engaged in DNA repair which makes their cells less sensitive than mouse cells to various stressors [152]. At the same time, when NMR fibroblasts were subjected to such damage-producing stressors, like: serum deprivation or hydrogen peroxide, they efficiently induced damaged cell elimination by apoptosis and autophagy [153].

Another reason for the long lifespan of NMRs is their apparent cancer resistance. Cancer incidence in these animals is very sparse, and it was thought for decades that these animals were fully cancer-resistant. The first two individuals with spontaneous tumors that developed at ages 20 and 22 were revealed quite recently. Histopathological examinations allowed us to identify these lesions as neuroendocrine carcinoma and adenocarcinoma, respectively [154]. Nonetheless, NMRs suffer from cancer incidentally, which is in keeping with their low sensitivity to form tumors even upon their engineered induction. Experiments on NMR-derived skin fibroblasts transduced with SV40 large T antigen and Ras/G12V showed that they were unable to generate tumors upon transplantation into animals in vivo, which differs from cells of mouse or rat origin that when similarly transduced formed tumors efficiently [155]. NMR cells possess an additional mechanism preventing excessive cell proliferation, termed early contact inhibition (ECI). ECI refers to the reaction of cultured NMR-derived fibroblasts that undergo contact-dependent growth inhibition—one of the significant anticancer mechanisms [156]—at a far lower density than mouse fibroblasts. Mechanistically, ECI has been found to depend on intact p53 and pRb tumor suppressors and the assistance of p16, which differs from the usual contact growth cessation in which the pivotal role is played by p27 [157]. A critical effector of ECI is the pALTINK4a/b protein, which is an additional product of the INK4a/b locus (apart from p15, p16, and ARF), but is absent in humans and mice [158].

Further research showed that ECI in NMR cells may be evoked by their high susceptibility to a specific form of ECM-derived hyaluronic acid (HA), the molecular weight of which is five times larger than that of its human or mouse counterpart [159]. This kind of HA is uniquely folded, and the pattern of folding is different in various tissues [160]. Experiments by Zhao et al. showed that 2D and 3D breast cancer microenvironments subjected to high-molecular-weight HA, similar to that produced by NMRs, were characterized by augmented apoptosis and inhibited proliferation of breast cancer cells, as well as by reduced tumor formation in nude mice in vivo. These anticancer effects of high-molecular-weight HA were attributed to the up-regulation of p53, followed by increased proapoptotic signals related to p21 and Bax [161]. Another mechanism by which NMRs may be protected against cancer is their effective immune system. A comparison of macrophages from NMRs and mice showed that the former display a lower increase in apoptosis and a higher degree of NF-κB induction and its downstream cytokine production upon stimulation [162]. Single-cell RNA sequencing showed that, conversely to cancer-prone mice, the NMR immune system exhibits a high ratio of myeloid-to-lymphoid cells, which may constitute its myeloid-based system of innate immunosurveillance, critical in eliminating cancer cells [163]. Another report based on NMR genome sequencing shows up-regulated *Smad 3* during NMR aging, the effect of which—taking into account that this molecule modulates antiproliferative TGF-β signaling—may point to the role of this alteration in optimizing the cell growth rate and protection of these animals against cancer [146].

One of the evolutionary traits that evolved to protect cells and organisms against cancer is cellular senescence [164]. The literature provides conflicting data regarding this phenomenon in NMRs. Experiments on SV40 TAg/Ras-expressing skin fibroblasts showed that they proliferate very well but after 40 divisions enter a crisis state, despite their transduction with oncogenes. Only the ectopic expression of the catalytic subunit of telomerase (hTERT) allowed the cells to surpass the crisis and proliferate further [155], which suggests that shortening or, at least, the stability of telomeres may be critical for their sustained proliferation. This is in agreement with a report by Zhao et al., who demonstrated the presence of oncogene-induced senescence in NMR fibroblasts transfected with HRasV12 plasmid [165]. These data are, however, in conflict with an observation that there is no age-associated shortening but the elongation of telomeres in leukocytes during NMR aging, which contrasts with the findings in rats and mice in which aging produces significant attrition of telomeric DNA [166]. This leads to the conclusion that replicative, division-driven senescence in NMR cells is negligible.

Conversely, NMR fibroblasts are prone to undergoing a stress-induced type of senescence, which was shown upon their treatment with DNA-damaging mitomycin C. Upon such exposure, the cells displayed enlarged and flattened morphology and increased expression of γ-H2A.X foci (a marker of senescence-associated DNA damage response) and SA-β-Gal. The induction of senescence correlated with the up-regulation of p16 and Arf, the probable effectors of cell-cycle arrest. Significantly, this induction of cell-cycle inhibitors was also present in cells forced to senescence by serial passaging, which denies to some extent the lack of replicative senescence in NMR cells [167]. If spontaneous replicative senescence in NMRs truly exists, it may be reminiscent of a so-called developmentally programmed senescence that was found in newborn NMRs. This kind of senescence that was evidenced according to the copresence of SA-β-Gal and p21 was abundant in various tissues of NMRs but at the same time generally absent or minimal in newborn mice. Senescence in NMRs was also achievable by exposure to γ-irradiation, and NMR cells appeared to be less vulnerable to the induction of stress-induced senescence than mouse cells, as they required a higher radiative insult to reach comparable subsets of senescent cells [165]. This difference may be the key for justifying the different lifespans of NMRs and mice based on their senescence-associated cell behaviors. At the same time, it must be stressed that, in contrast to the quite well-recognized issues of oxidative stress and cancer resistance in NMRs, the phenomenon of cellular senescence in these animals is still very elusive.

Last but not least, NMRs are organisms in which the extraordinarily long lifespan is determined by a large number of different but, to some extent, overlapping biological phenomena. This means that their longevity is determined by five out of six critical determinants, including cancer resistance, particular characteristics of cell divisions, a specific living environment, unique metabolic adaptations, and low damage to macromolecules. The list of major biological variables affecting aging dynamics and, under some circumstances, making it negligible also includes specific reproductive patterns.

## Blind mole rats

The blind mole rats (BMRs, genus *Spalax*) are subterranean rodents that, similar to their cousins, the naked mole rats are highly resistant to hypoxia [168] (e.g., through the downregulation of various energy-consuming liver function pathways [169] and increased blood vessel density [170]) and display exceptionally long lifespans. The maximum reported lifespan of NMRs was 21 years [171]. The next common feature of NMRs is their resistance to cancer. According to Gorbunova et al., there was no single incidence of spontaneous cancer in BMRs during 40 years of their observations [172]. BMRs are also very resistant to exogenous carcinogens, which differs from mice and rats in which these agents generate tumors very easily [173].

A possible explanation of this cancer resistance was provided by experiments on lung and skin fibroblasts isolated from lung and skin from two representative BMRs, *Spalax judaei* and *Spalax golani*. The experiments showed that these cells display uniform, vigorous proliferation, and in contrast to NMRs, they can reach high-density confluency without early contact inhibition (ECI), which is considered to be the prime barrier preventing excessive proliferation in NMRs [157]. Instead, BMR-derived cells were able to pass through a relatively small number of population doublings (7–20) and then began to release proinflammatory IFN-β and eventually died due to massive necrosis within 3 days. Because the occurrence of necrosis was abolished by targeting p53 and pRb by simian SV40 large T antigen, these two tumor suppressors emerged as critical for growth cessation of BMR fibroblasts [172]. Replicative senescence in BMR-derived fibroblasts was manifested by the cytosolic activity of SA-β-Gal and the overexpressed mRNA for p16, p21, and p53 [174]. Importantly, the termination of the replicative capabilities of these cells was not associated with any loss of telomeric DNA, plausibly due to the high activity of telomerase. The telomeres themselves in BMRs appeared to be very long, as they reached 50 kbp [172]. An effective maintenance of telomeric DNA integrity was also documented by Domankevich et al., who demonstrated that skin fibroblasts from *Spalax carmeli* accumulate lower amounts of DNA damage (histone γ-H2A.X) and have higher DNA repair capacity than fibroblasts of rat origin upon exposure to genotoxic stressors such as hydrogen peroxide, etoposide, UV-C, and hypoxia [175].

Very recent studies provided another explanation for BMR cancer resistance based on a unique feature of their cells. Namely, senescent fibroblasts appeared to be negative for one of the most procancerous traits seen in human somatic cells, which is the senescence-associated secretory phenotype (SASP) [174]. SASP refers to the ability of senescent cells to overproduce multiple cytokines (e.g., IL-1, IL-6), chemokines (e.g., IL-8, MCP-1, GRO-1, SDF-1), growth factors (e.g., TGF-β, VEGF, heregulin), and extracellular matrix (ECM) resysteming agents (e.g., PAI-1, -2, tPA, uPA) that promote various steps in tumor progression, including adhesion, proliferation, migration, invasion, epithelial–mesenchymal transition (EMT), and angiogenesis [176]. Senescent BMR cells possess undetectable or decreased expression of several SASP proteins, such as IL-6, IL-8, GRO-1, and ICAM-1, which indicates that in contrast to senescent human somatic cells [177], they are unable to support tumor growth [174].

## Bats as long-lived flying mammals

Bats (order: Chiroptera), the only mammals capable of powered flight, belong to the group of organisms with an exceptionally long lifespan, as they live even 10 times longer than predicted according to their body size [178]. The record holder bat is *Myotis brandtii*, who lived in the wild for more than 41 years [179]. Such a long lifespan of bats is a unique feature, as it occurs in animals with such a small body size and a superior metabolic rate [180]. Regarding their reproductive behavior, bats are very close to much larger, long-living mammals (e.g., elephants) having a small number of relatively sizeable neonates [181], which may suggest the presence of a trade-off between longevity and reproduction adhering to the disposable soma theory of aging. Flying abilities that restrict the risk of mortality related to predator fit, in turn, to life-history theory predicting that long lifespan is positively selected thanks to low extrinsic mortality [182]. Despite these findings, which are relevant from the perspective of bat evolutionary biology, a recent study by Wilkinson and Adams revealed that bat longevity is determined to the largest extent by their low body mass and periods of hibernation [183].

Some explanations for the long lifespan of bats also derive from their cellular physiology, particularly protection against oxidative stress and stability of telomeres. A comparative analysis of various tissues obtained from the short-lived *Myotis velifer* and the long-lived *Desmodus rotundus* showed that the latter display higher activities of the antioxidative enzymes SOD, CAT, and GPX and a lower degree of oxidative DNA injury [184], suggesting that bat cells are well protected against oxidative stress. Experiments on *Myotis lucifugus* showed that the generation of ROS per unit of consumed oxygen is lower than that in other mammals of a similar size, which denies the assumption that the high metabolic rate will simply translate to the high generation of ROS as byproducts of intensified mitochondrial respiratory chain reactions [185]. More likely, bat mitochondria developed some mechanisms that allow them to consume oxygen more efficiently, avoiding the risk of excessive ROS release. With regard to telomeres, they turned out to shorten during aging in *Rhinolophus ferrumequinum* and *Miniopterus schreibersii*, but not in long-lived *Myotis myotis* and *Myotis bechsteinii*, despite the lack of telomerase activity in the latter. Mechanistically, the maintenance of telomeres in *Myotis* bats is plausibly associated with the presence of 14 differentially expressed genes underlying DNA repair and 5 genes contributing to an alternative lengthening of telomeres (ALT) phenomenon [186]. Evidence for positive selection of genes responsible for telomere maintenance (*DKC1* and *TERT*) has also been revealed for *Myotis lucifugus* [187]. Interestingly, telomeres, the length of which appeared to display high variability from year to year, are prone to climate variables, including average temperature, minimum temperature, rainfall and windspeed. At the same time, the heritability of telomere lengths variance was minimal [188]. These findings indicate that telomere dynamics may be affected by external factors, which resembles observations on *Salmo trutta L.* (brown trout), in which telomere length was negatively correlated with average river temperature [189].

## Tortoises and their evolutionary predispositions for a long-lasting lifespan

Turtles belong to the longest-lived vertebrate animals. In particular, a long lifespan characterized giant tortoises common in the past on the western Indian Ocean islands and Ecuador Galapagos islands. According to various sources, these animals were able to live often far more than 150 years, such as Tu’i Malila (188 years old at death) [190] and Harriet (176 years old at death) [191].

The long lifespan of the giant tortoise corresponds to their low fertility level, which entirely adheres to both Williams’ (antagonistic pleiotropy [192]) and Kirkwood’s theories of aging predicting that a genetic investment in lifespan (possibly through a directed expenditure of energy towards soma maintenance [70]) occurs at the cost of reduced fertility and vice versa. Experimentally, this evolutionary trade-off was well described, e.g., by Rose et al., in *Drosophila melanogaster* in which an acceleration of mating and sexual reproduction resulted in shortened lifespan, whereas a delay in reproduction significantly extended the lifespan of the progeny [12].

The fertility of the giant tortoise is strongly linked with environmental conditions, particularly temperature and humidity. In the wild, they prefer to mate in the rainy period and nest in the dry period at the highest temperature [193]. In captivity, in turn, their reproductive behavior displays opposite characteristics: the animals mate when the environment is dry and hot and nest upon rains [194]. Branson et al. revealed that the low reproduction of the giant tortoise might depend on their specific dissociative reproductive pattern in which spermatogenesis temporarily passes through mating activity. In brief, captive Galapagos *Chelonoidis nigra* males displayed a negative correlation between testosterone levels and their mating activity. This hormone level was the lowest during the mating season and increased at the end of the nesting period. Reproductive activity was not synchronized even after a period of male and female physical separation.

Interestingly, female individuals did not exhibit signs of reproductive aging as they have sustained waves of ovarian follicular activity [195]. A similar phenomenon of a lack of reproductive output decline with age has been demonstrated by Congdon et al., who studied the oldest females of long-lived painted turtles (*Chrysemys picta*) [196]. Other observations made on Blanding’s turtle (*Emydoidea blandingii*) show that old individuals exhibit better survivorship and reproductivity than younger animals, which altogether suggests that reptiles do not fit with the fairly common senescence hypothesis of aging that predicts that reproduction or survival of older animals is reduced at the cost of their younger counterparts [197].

Another plausible explanation of giant tortoise longevity is the relatively slow metabolic rate that allowed them to survive on the small rations available on islands. Indeed, it has been shown that oxygen consumption decreases as the bodyweight of the Aldabra tortoise increases [198], which could also indicate that their long lifespan may be theoretically associated with decreased amounts of generated ROS [199]. This is, however, not a general rule, because in some species, the lifespan extension caused by decreased energy metabolism proceeds without a concomitant reduction in ROS [200].

A markedly depressed metabolic rate, up to 20% of the corresponding aerobic rate, is plausible as one of the mechanisms of the tortoise’s ability to tolerate anoxia and reoxygenation, which could plausibly also contribute to the longevity of these reptiles. The Trachemys and Chrysemys genera of freshwater tortoises are facultative anaerobes that can exist without oxygen for a long time [201]. A reduction in metabolism is then necessary to minimize their energy requirements and fuel their metabolic needs using exclusively the ATP derived from anaerobic processes. It has been demonstrated that tortoise brains, which are organs that are particularly sensitivity to depressed delivery of oxygen, have unique predispositions for anoxia, which are based on an equilibrium between excitation signals stabilized by dopamine and glutamate release and opposing inhibitory signals strengthened by intensified stimulation of GABA receptors. Anoxic brains also displayed improved reactions to ROS and oxidative DNA injury [202].

Additional adaptations to anoxia that have been recognized in *Trachemys scripta elegans* tortoises include high constitutive activity of antioxidants, such as SOD, CAT, and alkyl hydroperoxide reductase [203]; high tissue resources of total glutathione [204]; high expression of heat-shock proteins, e.g., Hsp72 [205], and inducibility of heat-shock transcription factor 1 (HSF1) and chaperones Hsp25, Hsp40, Hsp70, Hsc70, and Hsp90 [206]; and an NF-κB-dependent increase in the transcription of anti-apoptotic Bcl-2 and Bcl-x_L_ proteins [207].

The results of the quite recent genome sequencing of the Pinta tortoise “Lonesome George”—the last representative of the Galapagos Islands native *Chelonoidis abingdonii* [208], who died at an estimated age of more than 100 years old—and the “Aldabra” giant tortoise (*Aldabrachelys gigantea*) living on the remote Aldabra Atoll shed more light on the unique physiology of giant tortoises. This research showed that the genomes of these reptiles, in contrast to various shorter lived vertebrates, display multiple copies of several groups of genes that could be treated as positively selected by evolution as a genetic base of their extraordinary lifespan [209]. There was a multiplication of immune system-related genes, including the *PRF1* gene coding for perforin and the *APOBEC1*, *CAMP*, *CHIA*, and *NLRP* genes, which are involved in host response reactions against viral, microbial, fungal, and parasite infections, respectively. Expanded genes also included putative tumor suppressors, including *SMAD4*, *NF2*, *PML*, *PTPN11*, and *P2RY8,* which could correspond to the very rare frequency tumors found in tortoises [210]. The giant tortoise genome also contained multiple copies of genes responsible for the maintenance of genome integrity, such as *NEIL1*, *RMI2*, and *XRCC6*, contributing to base-excision repair, DNA break end resection, and helicase activity, respectively [209].

The last portion of gene multiplication providing some protection for DNA is intriguing from the perspective of the role of telomeres in tortoise aging. Studies on white blood cells isolated from the wild giant leatherback turtle *Dermochelys coriacea* showed that telomeres in these cells do not shorten with age, which was supposed (but not proved experimentally) to have high telomerase activity. Interestingly, the authors of the study showed that telomeres were shorter in females breeding after a 2-year migration than in those that migrated a year longer. According to the authors, these differences may indicate that blood telomeres were in better shape in those individuals who restored their body (energy?) reserves for a longer time (at the cost of earlier reproduction) than in those in whom breeding occurred more frequently [211]. This association of body maintenance, a slower rate of telomere attrition, and lower reproductive output is additional evidence pointing to the correctness of the evolutionary theory of antagonistic pleiotropy. A lack of telomere shortening in white blood cells during aging was also shown in captive loggerhead tortoise (*Caretta caretta*) [212], which may suggest that cellular mechanisms involved in the aging of tortoises do not include damage to telomeric DNA, perhaps due to the aforementioned genetic predispositions [209]. At the same time, skin fibroblasts from young Galapagos tortoises have been found to proliferate far more vigorously and reach longer lifespans than fibroblasts from old animals. More importantly, the number of achievable divisions by tortoise cells (100–130) was higher compared with humans (< 100), which may imply that organismal lifespan in vivo is to some extent reflected by the dynamics of somatic cell senescence in vitro [213]. An even higher number of doublings were reported in cells from the long-lived snapping tortoise (*Chelydra serpentina*) that appeared to be able to go through as many as 265 divisions. This long replicative lifespan could be associated with a specific pattern of telomerase activity. Namely, the level of this enzyme in cells at 157 population doublings was barely detectable, as evidenced using a radioactive isotope-based TRAP assay. Then, however, it started to increase progressively until reaching a very strong signal in cells at 191 divisions. Importantly, however, high activity of telomerase may be not the sole explanation of *C. serpentina* longevity*.* A continuous, unchanged proliferation rate of cells at 265 may suggest the presence of a specific cell replacement mechanism in which some cells are replaced by their immortal counterparts that do not display any signs of malignant transformation or senescence [214]. Profound telomerase activity was also found in old painted turtles (*Chrysemys picta*), whose cells can replicate up to 120 population doublings [215]. It is also likely that an exceptionally long lifespan of tortoises may be associated with long telomeres in their cells. Indeed, it has been found that telomere length in *Pseudemys scripta* was approximately 50 kbp [216] and was even above 60 kbp in *Chrysemys picta* [215], which are impressive values compared with normal human cells (e.g., < 15 kbp in lymphocytes) [217].

## Elephants and their longevity

The maximum lifespan for wild female African elephants (*Loxodonta africana*) and Asian elephants (*Elephas maximus*) has been estimated at 74 [218] and ~ 80 years [219], respectively. Large body size and a lack of natural enemies and predators, both limiting the probability of environmental death, are considered the driving forces of the relatively long lives of these animals. Paradoxically, captivity appeared to increase mortality and shorten the lifespan (vs. captive-born animals) of elephants, as evidenced by analysis of the life-histories of over 5000 captive Asian elephants [220]. This observation is exciting as it challenges a classic dogma that protection from natural hazards provided by humans in zoos or laboratories prolongs animal lifespan and thus induces aging in those species in which this phenomenon was negligible in the wild due to high external mortality [221].

External conditions are, however, important for reproductive aging of these animals and late-life fitness that apparently declines [222], analogously to humans and other long-lived mammals. Observations made on Asian elephants showed that females born in stressful conditions generated by high workloads for their working mothers and the monsoon season (established according to increased glucocorticoid metabolite levels) undergo accelerated reproductive aging and diminished reproductive output compared with animals born in nonstressful periods of the year. This, in turn, indicates that early conditions experienced by an animal (mother) may significantly affect the reproductive aging of its progeny, which could be explained as a result of different requirements regarding an investment of the mothers in their soma maintenance (e.g., if additional energy resources are needed to overcome unfavorable conditions) [223]. From an evolutionary point of view, it also adheres to observations by Hayward et al., who showed the relationship between early life reproduction in Asian elephants and impaired later life survival [224]. At the same time, these results conflict with research on wild African elephants in which no differences in survival were noted between females that started reproducing earlier and those who commenced reproduction later [225].

Similar to naked mole rats, whose long-lasting lives are linked to some extent with a low incidence of cancer, elephants are also known as animals that very rarely get cancer. It has been estimated that cancer mortality in elephants (both African and Asian) accounts for 4.81%, which is significantly lower compared with humans, in whom this value reaches 11–25% [226]. This cancer-resistant phenotype of elephants is linked with their unique genetic, cancer-protecting profile, which is often given as an explanation of the so-called “Peto’s paradox” [227]. This means that elephants that are characterized by either large size and long lifespan do not display a robust cancer incidence, as would be expected based on the assumption that larger organisms (due to a greater number of cells) and longer life generate more cellular/DNA targets and the time available for the occurrence of procancerous mutations. A positive correlation between body size/lifespan and cancer frequency that has been shown in various species, e.g., dogs [228] or humans [229], is absent when different species are compared [226].

With regard to African elephants, sequencing of their DNA revealed that it contains 20 copies of the p53 tumor suppressor (vs. 1 copy in humans), which indicates that their genes are exceptionally well protected by this guardian of the genome. Specifically, only one of these 20 copies was similar to that of human origin; the remaining 19 copies have been recognized as retrogenes, which are intron-free products of a reverse conversion of RNA to DNA. Moreover, apoptosis driven by p53 in lymphocytes from African and Asian elephants in response to DNA injury occurred at a markedly higher rate than in cells of human origin, suggesting that quality control of DNA in these mammals may be more efficient than in representatives of *Homo sapiens* [226]. These observations have been strengthened by a recent report by Vazquez et al., who found that elephants bear another unique tumor suppressor, leukemia inhibitory factor pseudogene (LIF6), the transcription of which is up-regulated by p53 upon DNA damage, eventually leading to mitochondrial, Bax/Bak-dependent apoptosis [230]. Induction of apoptosis is also initiated by p53 retrogenes, which appear to contribute to the increased sensitivity of elephant DNA to damage. Significantly, proapoptotic signals in elephants are sent at a much lower degree of DNA injury than in other mammals, suggesting that mutation removal occurs at very early stages of tumorigenesis [231].

The longevity of elephants may also be connected with the stability of their telomeres, particularly the rate of their shortening. Comparative studies on various species, including the Sumatran elephant (*Elephas maximus sumatranus*) using the high-throughput quantitative fluorescence in situ hybridization (HT Q-FISH) method allowing analysis of telomeres at a single-cell level, showed that the rate at which telomeres are shortened in elephant cells (109 bp per year) is slower compared with mice (6420 bp per year), Bottlenose dolphins (766 bp per year), goats (363 bp per year), reindeer (531 bp per year), griffon vultures (209 bp per year), and Audouin’s gulls (771 bp per year). At the same time, the authors of this study did not reveal any correlation between species lifespan and initial telomere length, which may imply that the magnitude of DNA injury and/or efficiency of the repair mechanism play a significant role as determinants of an organism’s lifespan [232].

## Killer whale as an example of post-reproductive aging

In a classic evolutionary paradigm, aging refers to the post-reproductive period of life and thus it applies to only a small groups of animals in nature [221]. This view has changed in recent years, since several long-term field studies provided mounting evidence that aging is commonly detected in nature [233]. Regardless of these divergent views, the biology of the killer whale (*Orcinus orca*) is an excellent example of the so-called post-reproductive aging. This means that these animals, similarly to humans, manifest a prolonged post-reproductive life span: a relatively long period of a female’s life after reproduction where she has no ability to reproduce any further [234]. Precisely, female killer whales cease to reproduce in their late third and early fourth decade of life, but can continue to live for several decades afterward [235]. When it comes to the post-reproductive aging phenomenon in humans, the most plausible explanation includes benefits that post-reproductive females can provide by assisting their relatives (so-called grandmother hypothesis) [236]. Interestingly, a similar situation has been described in the case of killer whales in which grandmothers experiencing the post-reproductive period of life increased the survival of their grandoffspring [235]. Another evolutionary determinant of long post-reproductive life in killer whales may be the fact that a high ratio of females (relative to natural populations of most species) live to advanced age, therefore boosting selection on late-life effects [237]. There is also another hypothesis, termed “reproductive conflict hypothesis” which assumes that younger females that invest more in rivalry have greater reproductive success than their older counterparts (mothers) when reproducing at the same time [238].

## Organoids as an alternative tool for research on cellular and systemic aging

Organoids belong to the youngest experimental tools with a high capacity to serve as a valuable system of aging in the next decades. From a semantic point of view, the term *organoid* refers to a human-produced, three-dimensional (3D) construct, the organization of which closely recapitulates the histological architecture of human tissues in vivo and provides an opportunity to use it as an artificial organ equivalent for in vitro studies of a wide array of biological phenomena. Organoids constitute the next step in cell culture methodology in which some of the cons of conventional two-dimensional cell culture systems (e.g., monocellularity, lack of cell interactions with stromal proteins, cell growth on plastic surfaces) are significantly improved or eliminated. Most frequently, 3D organoids consist of one or more cell types that physiologically create an original tissue and adjacent acellular constituents, mainly extracellular (ECM) proteins [239]. The principle in creating reliable organoids is to reflect an exact histology and tissue organization as far as possible [240]. In recent years, experiments using organoids have been seriously treated as an alternative for preclinical research on experimental animals [239] and are of particular interest in oncological studies [241].

Recently, organoids were successfully used to system epigenetic changes associated with the aging of human intestines, which was based on a more general prediction that the DNA methylation profile allows the quantification of aging progression at the individual [242] and tissue levels [243]. In this context, Lewis et al. strengthened the above predictions by showing that duodenal spheroids display DNA methylation aging rates that are comparable with the uncultured epithelial crypts from which the spheroids were established, although their epigenetic age was slightly reduced compared with chronological aging. In colon-derived spheroids, the DNA methylation age was also comparable with the source crypts; however, the epigenetic aging in these spheroids closely matched the chronological age of the donor. All these data suggest that chronological aging is preserved in cultured, organoid-based conditions at the epigenetic level, albeit some regional variations exist [244]. Organoid cultures established from primary multipotent cells were also used to examine the genome-wide mutation profile in stem cells from the small intestine, colon, and liver in donors varying in age. The results showed that the number of mutations rises progressively with age in all of the tested organs, and the pace at which the lesions accumulated was estimated at 40 new mutations per year [245]. Intestinal epithelial organoids derived from both young and aged mice demonstrated that senescence of intestinal cells is associated with the accumulation of SA-β-Gal and DNA demethylation-dependent up-regulation of the cell-cycle inhibitor p21. This system also revealed that the decreased proliferation of the cellular fraction of organoids from aged mice results from histone H3 lysine 27 trimethylation-related epigenetic silencing of the stem cell marker *Lgr5* and the resulting suppression of Wnt signaling [246]*.* Another kind of organoid from the mouse colon and human small intestine and colon was used to demonstrate that the addition of recombinant Wnt3A to the combination of growth factors applied to mouse colon crypts allowed them to avoid the Hayflick limit and to replicate indefinitely. Long-term culture of human cells was achievable upon introduction of inhibitors of p38 MAPK and ALK to the system [247], proving that premature senescence of somatic cells may be elicited not only by inadequate culture conditions [248] but also by hyperactivity of some strictly defined internal signaling pathways.

Lozito and colleagues describe the design of a 3D osteochondral microsystem to investigate osteoarthritis, one of the most common age-dependent degenerative diseases. The system is intended to avoid limitations resulting from separate tests of the cartilage or the bone component of the articular joint and to provide an opportunity to examine osteochondral integrity and behavior. The whole system originates from mesenchymal stem cells isolated from bone marrow or from adipose tissue that differentiate into bone, cartilage, and synovial sections of the microtissue [249]. Because cartilage stiffening resulting to a large extent from the accumulation of glycation end-products (AGEs) plays a pivotal role in osteoarthritis [250], 3D noncellular collagen matrix has been used to demonstrate that glycation of collagen increases the stiffness of the matrices and generates AGEs, and the efficiency of these processes depends on the concentration of ribose that was used as the glycating agent. Apart from changes in the mechanical properties of collagen, the glycated matrices affected the viability, growth, and some functional features (e.g., differentiation into a contractile phenotype) of fibroblasts [251]. 3D bioprinting technology linking poly(ethylene glycol) dimethacrylate with human chondrocytes was successfully used to repair deficiencies in osteochondral plugs, proving the potential of cartilage engineering using 3D artificial microtissues [252].

Another target for organoid employment is age-associated neurodegeneration, e.g., Alzheimer’s disease (AD) [253]. To date, both the pathogenesis and experimental pharmacology of AD have mainly focused on animal systems and cultures of nonneuronal cells, and for this reason, not all critical aspects of neuropathology may be adequately reflected [254]. Promising results have been derived from tests on human neural stem cell-derived 3D organoids in which the accumulation of amyloid β (Aβ) plaques and neurofibrillary tangles was achievable. The pathogenic traits of AD were induced by multiple mutations in amyloid precursor protein (APP) and presenilin 1 (PS1) neural stem cells, which allowed the generation of significant amounts of 42-residue Aβ, comparable to those in the brains of patients with AD [255]. In contrast to conventional 2D culture systems in which Aβ diffuses into a culture medium, the 3D Matrigel system of AD allows the collection of the whole secreted amyloid, allowing its aggregation. The organoid also contained deposits of hyperphosphorylated tau proteins. The usefulness of this system to the pathogenesis of AD in accord with the amyloid theory of the disease was tested in experiments in which the inhibition of Aβ formation and tau protein phosphorylation was effectively prevented by exposure to β- and γ-secretase inhibitors [256].

Recently, successful attempts have been made to merge organoid and organ-on-a-chip technology to create complex multilayer tissue systems in a human retina-on-a-chip platform [257]. The system contains more than seven different essential retinal cell types originating from human induced pluripotent stem cells and vasculature-like perfusion, which allows its use in research on retinal diseases, including age-related macular degeneration (AMD). As per research on early changes occurring during AMD development, another kind of 3D organoid was constructed based on a polycaprolactone-gelatin electrospun scaffold combined with human retinal pigment epithelial cells and primate choroidal cells [258]. Reciprocal interactions between retinal pigment epithelium and choroidal endothelial cells, which are two types of cells whose damage directly leads to AMD, can be tested in turn on a bilayer coculture system placed on transwell inserts in which the disease conditions are mimicked by exogenous VEGF-driven permeability of the bilayer [259].

## Concluding remarks

The history of research on aging, longevity, and immortality is full of paradoxes. One of the most striking is the fact that August Weismann, the father of the wear and tear theory of aging, which, although valid to a large extent (the process occurs, because somatic cells cannot renew themselves [260]) but for him unverifiable, was the same person who originally described in 1883 an immortal organism, *Turritopsis dohrnii* [261]. Indeed, since his discoveries made in the XIX century, a significant number of systems of aging have emerged, which translated to only partial insight into this phenomenon. On the other hand, one must admit that although we did not reach an extension of maximum human life span despite extensive research in the area of aging, we do experience a progressive increase in the average length of human life (life expectancy). And, even more importantly, this trend is accompanied by a similar extension of the period of healthspan (healthy old age), which primarily stems from the development of medicine and pharmacology reducing late-life mortality [262].

This review of non-conventional systems of aging provides some conclusions that should be considered from a broader and more general perspective. The first is that mechanisms of aging, longevity, and immortality can be grouped into six major categories that include several detailed issues associated with: (1) cell divisions, regeneration, and senescence; (2) cancer resistance; (3) metabolism and specific adaptations; (4) environmental conditions; (5) reproduction-associated features; and (6) quality control and damage to macromolecules (Table 2). Importantly, however, the proportions between detailed mechanisms belonging to each group significantly differ between species. Some of them, e.g., adaptations to suboptimal environmental conditions, oxidative stress, accumulation of abnormal proteins, stability of DNA, efficiency of DNA repair, and maintenance of telomeres, appear to be widely distributed and conserved among species of different phylogeny. On the other hand, some determinants, such as regenerative capacity associated with stem cell activity, specific pattern of reproduction, or lack of natural enemies, seem to play a role only in a narrow group of organisms. This observation leads to the second conclusion which may indicate that the “true” and universal reason(s) of aging probably belongs to the group of the most conserved pathways and phenomena. Is this assumption true? Some may argue that it is not because the picture of aging differs too much between various species. In our opinion, however, the mechanistic background of aging may be to some extent universal, whereas the reasons of longevity and immortality seem to be more sophisticated and species-specific.

**Table 2:** Six major groups of biological determinants of aging, longevity, and immortality found in research on less conventional experimental systems

Mechanism/pathway	Experimental system
Cell divisions, regeneration, senescence	
Telomeres and telomerase	*T. dohrnii; A. islandica; N. furzeri; H. glaber*; *S. judaei; M. brandtii; M. lucifugus;* giant tortoises; Sumatran elephant
Specific regulation of cell cycle (e.g., early contact inhibition)	*H. glaber; S. judaei*
Morphallaxis	*H. vulgaris*
Activity of stem cells	*T. dohrnii; H. vulgaris; H.* *oligactis*
Cell death (apoptosis, necrosis)	*A. islandica; H. glaber; S. judaei; L. africana*
Mutations	*C. crescentus*
Regeneration capacity	*H. vulgaris*
Cancer resistance	
DNA damage and repair	*H. vulgaris; H. glaber; S. carmeli; M. brandtii;* giant tortoises; *L. africana*
Multiplicated tumor suppressors	giant tortoises; *L. africana*
Retrogenes	*L. africana*
Specific molecules (high-weight hyaluronic acid)	*H. glaber*
Lack of SASP	*S. carmeli*
Metabolism and specific adaptations	
Slow metabolic rate	*A. islandica; H. glaber;* giant tortoises
Hibernation	*M. brandtii*
Hypoxia/anoxia, reoxygenation tolerance	*A. islandica*; *H. glaber; S. judaei;* giant tortoises
Environmental conditions	
Starvation	*E. coli; H. vulgaris; N. guentheri*
Stressful living conditions (e.g., suboptimal temperature, humidity, monsoons)	*E. coli; C. crescentus; T. dohrnii; H. vulgaris;* *H. oligactis*; *N. guentheri*; *N. furzeri; S. microcephalus;* giant tortoises; *L. africana;* Asian elephants
Flying capacity	*M. brandtii*
Lack of predators	*S. microcephalus; L. africana*
Living underground	*H. glaber*
Reproduction-associated features	
Low fecundity	*N. guentheri*; *M. brandtii;* giant tortoises; *L. africana*
Assymetric divisions (e.g., new and old poles)	*E. coli*
Specific reproduction cycles (budding, polyps, swarmer and stalked cells)	*C. crescentus; T. dohrnii; H. vulgaris; N. guentheri*
Hormones	giant tortoises
Gametogenesis	*T. dohrnii; H. oligactis*
Quality and damage to macromolecules	
Aggregated proteins	*E. coli; C. crescentus*; *N. rachovii; S. microcephalus; H. glaber *
Oxidative stress, ROS, antioxidants	*E. coli; H. vulgaris; A. islandica*; *N. guentheri*; *N. furzeri*; *S. microcephalus; H. glaber; M. brandtii;* giant tortoises
Chaperones	*N. furzeri*
Mitochondrial metabolism	*N. furzeri; H. glaber*
Proteasome	*N. rachovii; H. glaber*
Autophagy	*H. oligactis*; *N. rachovii*

Detailed informations regarding each mechanism/pathway (e.g., its augmentation or inhibition) are provided in the text

Last but not least, the third and the most important conclusion which stems from research on the non-conventional systems is that they deliver multiple unique adaptations, metabolic traits, and cellular features that are highly specific to their biology and probably associated to the greatest extent with the course of their aging. The unanswered question remains if these phenomena may have some equivalents in humans or other more popular species. Factors that deserve the utmost attention include among others: hormetic-like up-regulation of antioxidants accompanying increased formation of ROS in hypoxic conditions, high content of free protein thiol groups [149], and high level of peroxidation-resistant membrane fatty acids [263], supporting the membrane pacemaker theory of aging [264], all observed in NMRs. At the same time, NMRs are characterized by specific early contact inhibition that restricts excessive cell growth and tumorigenesis. Their cousins, BMRs, may prolong their lives by inhibiting tumorigenesis using a unique IFN-β-dependent necrosis of cells. To the same category, one may also include the lack of SASP in senescent cells from BMRs, multiplicity of genes associated with DNA repair, and telomere stability in bats and those involved in immune system functioning in giant tortoises or several copies of the p53 tumor suppressor typical for some elephants. Observations of species belonging to the less frequent systems in biogerontology also showed the significance of low temperature as the physical variable determining longevity. In NMRs, declined body temperature represents an adaptation to living underground in hypoxic conditions. Low temperature may also matter in case of Greenland sharks which spend their longstanding lives 2000 m down, where the water temperature is between − 1 and 10 ºC. A hibernation-associated decrease in body temperature could be linked with relatively long lifespan of bats. Beneficial, life-extending impact of low temperature was also found in research on *N. furzeri.* Research on *T. dohrnii* and *Hydra* polyps provides irrefutable evidence of the importance of stem cells in longevity. And last but not least, experiments on such specific organism like *Hydra* provide a unique opportunity to make direct genetic and functional comparisons between non-aging and induced aging organisms. At the same time, studies using *N. furzeri* may generate increasing number of valuable data regarding ecological determinants of aging, as they represent an ideal combination of short lifespan, long stage of embryonic arrest, and high propensity to manipulations within environmental variables. They also display several vertebrate-specific features that are missing in another non-vertebrate organisms [265]. For example, research using this system has provided quite recently an insight into the role of the gut microbiota as a lifespan-modulating agent [106]. Taking all these facts into account, we believe that scientific investigations using various non-conventional systems of aging may provide several benefits for contemporary biogerontology and foremost open some new avenues to understand even better the mystery of this phenomenon.
